# Using Social Listening Data to Monitor Misuse and Nonmedical Use of Bupropion: A Content Analysis

**DOI:** 10.2196/publichealth.6174

**Published:** 2017-02-01

**Authors:** Laurie S. Anderson, Heidi G Bell, Michael Gilbert, Julie E Davidson, Christina Winter, Monica J Barratt, Beta Win, Jeffery L Painter, Christopher Menone, Jonathan Sayegh, Nabarun Dasgupta

**Affiliations:** ^1^ GlaxoSmithKline Research Triangle Park, NC United States; ^2^ Gyra MediPharm Consulting Research Triangle Park, NC United States; ^3^ Epidemico, Inc Boston, MA United States; ^4^ GlaxoSmithKline Stockley Park, Middlesex United Kingdom; ^5^ National Drug and Alcohol Research Centre, UNSW Australia Randwick Australia; ^6^ Bluelight.org Dover, DE United States; ^7^ Kadiant Analytics Boston, MA United States

**Keywords:** social media, Internet, prescription drug misuse, substance-related disorders, pharmacovigilance, harm reduction, community-based participatory research, bupropion, amitriptyline, venlafaxine hydrochloride

## Abstract

**Background:**

The nonmedical use of pharmaceutical products has become a significant public health concern. Traditionally, the evaluation of nonmedical use has focused on controlled substances with addiction risk. Currently, there is no effective means of evaluating the nonmedical use of noncontrolled antidepressants.

**Objective:**

Social listening, in the context of public health sometimes called infodemiology or infoveillance, is the process of identifying and assessing what is being said about a company, product, brand, or individual, within forms of electronic interactive media. The objectives of this study were (1) to determine whether content analysis of social listening data could be utilized to identify posts discussing potential misuse or nonmedical use of bupropion and two comparators, amitriptyline and venlafaxine, and (2) to describe and characterize these posts.

**Methods:**

Social listening was performed on all publicly available posts cumulative through July 29, 2015, from two harm-reduction Web forums, Bluelight and Opiophile, which mentioned the study drugs. The acquired data were stripped of personally identifiable identification (PII). A set of generic, brand, and vernacular product names was used to identify product references in posts. Posts were obtained using natural language processing tools to identify vernacular references to drug misuse-related Preferred Terms from the English Medical Dictionary for Regulatory Activities (MedDRA) version 18 terminology. Posts were reviewed manually by coders, who extracted relevant details.

**Results:**

A total of 7756 references to at least one of the study antidepressants were identified within posts gathered for this study. Of these posts, 668 (8.61%, 668/7756) referenced misuse or nonmedical use of the drug, with bupropion accounting for 438 (65.6%, 438/668). Of the 668 posts, nonmedical use was discouraged by 40.6% (178/438), 22% (22/100), and 18.5% (24/130) and encouraged by 12.3% (54/438), 10% (10/100), and 10.8% (14/130) for bupropion, amitriptyline, and venlafaxine, respectively. The most commonly reported desired effects were similar to stimulants with bupropion, sedatives with amitriptyline, and dissociatives with venlafaxine. The nasal route of administration was most frequently reported for bupropion, whereas the oral route was most frequently reported for amitriptyline and venlafaxine. Bupropion and venlafaxine were most commonly procured from health care providers, whereas amitriptyline was most commonly obtained or stolen from a third party. The Fleiss kappa for interrater agreement among 20 items with 7 categorical response options evaluated by all 11 raters was 0.448 (95% CI 0.421-0.457).

**Conclusions:**

Social listening, conducted in collaboration with harm-reduction Web forums, offers a valuable new data source that can be used for monitoring nonmedical use of antidepressants. Additional work on the capabilities of social listening will help further delineate the benefits and limitations of this rapidly evolving data source.

## Introduction

### Background

The nonmedical use of pharmaceutical products has become a significant public health concern [1]. The National Survey on Drug Use and Health (NSDUH) reported that in 2014, there were 6.5 million people aged 12 years and older in the United States who had used prescription psychotherapeutic drugs nonmedically in the previous month [2]. Although the focus of traditional surveillance systems is on controlled substances, concerns occasionally arise over patient-initiated nonmedical use of noncontrolled pharmaceutical substances and the potential associated morbidity and mortality [3]. These concerns go beyond nonadherence to recommended dosages, escalating to the use of drugs to experience psychotropic effects, and in combination with controlled substances to enhance euphoria or mitigate withdrawal.

An example is bupropion, a noncontrolled medicine approved in many countries for the treatment of depression and as an aid to smoking cessation. In early preclinical studies, bupropion showed amphetamine- and cocaine-like behavioral effects in animals [4-7]. However, human abuse potential studies determined that bupropion had lower abuse liability than amphetamine, methylphenidate, or caffeine when taken orally [8-11], which is the only approved route of administration. This research led to the noncontrolled classification of bupropion in the United States and elsewhere. More recently several published case reports of the nonmedical use of bupropion have emerged [12-18], with particular focus on criminal justice and prison settings [19-24]. In 2014, after reviewing several reports, GlaxoSmithKline updated the prescribing information, alerting clinicians to the risks of nonoral routes of administration [25]. A 2013 evaluation of the Drug Abuse Warning Network (DAWN) database to examine the number of reports for bupropion stratified by demographics, route of administration, and disposition of the patient during the study period 2004-2011 did not provide evidence that misuse and nonmedical use of bupropion was growing over time [26].

For newly marketed drugs that are suspected or confirmed to have misuse and abuse potential, traditional methods for pharmacovigilance signal detection include evaluation of spontaneous postmarketing reports; retrospective studies of observational databases, such as vital statistics and poison center calls; data from national surveys; surveys from surveillance systems that measure rates of abuse, misuse, and diversion, such as the Researched Abuse, Diversion and Addiction-Related Surveillance (RADARS) system; focused studies in geographic regions of interest; and literature reports. However, those who utilize prescription products nonmedically, for psychotropic effects, may be hesitant to report this use to health care providers, drug companies, and regulatory agencies, even when adverse events are experienced. In addition, traditional pharmacovigilance tools such as spontaneous adverse event reports, medical literature, observational databases, and national surveys have inherent time lags for data availability, often lack product specificity, and may not be specifically tailored for data collection on drug abuse [27].

With ease of access and instant feedback, more consumers are turning to social media (forms of electronic interactive media through which users create online communities to share information, ideas, personal messages, and other content) to discuss their medical experiences and ask questions about medications in general [28,29]. Others have proposed using social media data (Web forums; social network sites such as Facebook, Twitter, Instagram, and You Tube; blogs; and chat rooms) to support research findings [30-32], to conduct surveys [33-35], and for surveillance of pharmaceutical and illicit products [36-42]. Similar to the methodology of this study, some have also utilized social media specifically to evaluate the nonmedical use of prescription drugs [43-50]. Each of these studies used lexicon-based strategies for gathering social media content and qualitative analyses to identify perceptions and behaviors relating to nonmedical use of controlled pharmaceutical drugs. Although elements of the study designs reported in previous publications are similar to this study, they all focused on drugs with recognized abuse liability. However, this study focused on drugs that are neither controlled nor recognized by regulatory authorities as exhibiting abuse liability.

Conversations about nonmedical drug use do occur in harm-reduction Web forums. These websites first began to appear in the 1990s and are used to seek drug-related information, to share drug experiences with like-minded others, to reduce harm, to seek support, and to build a sense of belonging to a community, although often through participants using a pseudonym [51,52]. These functions of Web forums are particularly salient to people who are concerned about the social and legal ramifications of revealing illicit behaviors or stigmatized identities to their immediate personal networks [52]. The pseudonymous nature of Web forum identities sets them apart from those on the newer social media platform, Facebook, which has a “real name” policy [53]. Although many other Web-based communication platforms have been superseded in the Facebook era, pseudonymous Web forums in which drugs are discussed continue to retain existing communities and attract new members.

Social listening, in the context of public health sometimes called Infodemiology or infoveillance [54], is the process of identifying and assessing what is being said about a company, product, brand or individual, within forms of electronic interactive media [38,55]. The objectives of this study were (1) to determine whether content analysis of social listening data could be utilized to identify posts discussing potential misuse or nonmedical use of bupropion and 2 comparators, amitriptyline and venlafaxine, and (2) to describe and characterize these posts.

### Study Medication: Bupropion (Wellbutrin, Wellbutrin XL, Wellbutrin SR, Zyban)

Bupropion, a reuptake inhibitor of norepinephrine and dopamine, was approved by the United States Food and Drug Association (FDA) for the treatment of major depressive disorder in 1985 [25] and for the treatment of nicotine dependence as an aid to smoking cessation in 1997 [56]. Controlled clinical trials were conducted in normal volunteers, in subjects with a history of multiple drug abuse, and in depressed subjects. These studies showed some increase in motor activity and agitation or excitement, which is often typical of central stimulant activity. Evidence from single-dose trials suggests that the recommended daily dosage of bupropion, when administered orally in divided doses, is not likely to be significantly reinforcing to amphetamine or central nervous system stimulant seekers. Higher doses, which could not be tested because of the risk of seizure, might be modestly attractive to those who use the central nervous system drugs nonmedically. Stimulant adverse reactions reported from clinical trials include central nervous system stimulation and hypomania, and those reported from postmarketing include euphoria, hallucinations, and manic reaction [25]. Reports in the literature indicate cocaine-like high, stimulant high, and euphoric effects with bupropion [22,24,57].

### Comparator Medications

#### Amitriptyline (Elavil, Endep)

Amitriptyline is a tricyclic antidepressant (TCA) with known sedative properties and was approved by the FDA for the relief of symptoms of depressive illness in 1961 [58]. Sedative adverse reactions reported with TCAs include drowsiness, fatigue, disorientation, confusional states, and disturbed concentration [58]. There are discussions in the literature, including case reports, regarding the nonmedical use of amitriptyline [59-63]. The majority of case reports do not identify the route of misuse administration. When reported, the medications were described as taken orally, and in some cases, in large doses to produce a “euphoria” and a “pleasant” feeling [3,64].

#### Venlafaxine (Effexor, Effexor XR)

Venlafaxine is a serotonin and norepinephrine reuptake inhibitor (SNRI) and its extended-release formulation was approved by the FDA in 1997 for major depressive disorder, generalized anxiety disorder, social anxiety disorder, and panic disorder [65]. In clinical studies, there was no indication of drug-seeking behaviors; however, venlafaxine has not been systematically studied in clinical studies for its potential for nonmedical use. The United States prescribing information suggests that physicians carefully evaluate patients for history of nonmedical use of drugs and follow them closely for misuse or nonmedical use. Dissociative adverse reactions reported from clinical trials include sweating, dizziness, hallucination, and depersonalization; postmarketing reports include delirium [65]. There are case reports in the literature that describe large doses of oral ingestion (4050 mg and up to 3750 mg/day) to achieve altered states (“amphetamine-like high,” “more empathic and sociable,” and “elated” mood) [66-67]. These cases suggest that the nonmedical use of SNRIs may result in amphetamine-like effects or the dissociative effects of excess serotonin [3].

## Methods

### Study Design

This was a retrospective, observational, and qualitative content analysis [68]. We analyzed all cumulative data on 3 noncontrolled antidepressant drugs (bupropion plus two comparators, amitriptyline and venlafaxine). As minimal work has been done in evaluating the nonmedical use of noncontrolled substances, comparator antidepressant data provided context in evaluating outcomes. Amitriptyline and venlafaxine were selected as comparators because they are indicated for depression; however, each one represents a unique mechanism of action for effect. In addition, similar to bupropion, the United States regulatory approvals of amitriptyline and venlafaxine predate the existence of the two target Web forums, thus increasing the chances of seeing the discussions reflecting these drugs. Summary statistics on numbers of posts, threads, and authors for 4 additional controlled substances (methylphenidate, alprazolam, buprenorphine, and oxycodone) were also collected and compared for contextualization.

### Data Sources

Data were collected from two publicly available harm-reduction forums (Bluelight and Opiophile) from their launch dates (1997 and 2003, respectively) through July 29, 2015. The sites were chosen from pilot work suggesting that these were particularly rich databases for this type of information. Bluelight has been in operation continuously since 1997, and is the largest global drug discussion website with over 320,000 members and nearly 7 million posts. Opiophile, in contrast, has experienced several periods of downtime since its launch in 2003, owing to server issues. At the time of data analysis, it had been offline since mid-2015, and had only 7927 members and just fewer than 100,000 posts at study inception.

### Data Processing

Message preparation began with extracting a set of generic, brand, and vernacular product names, including misspellings, from Epidemico’s MedWatcher Social product dictionary [38,69]. That set of product names was then used to identify posts. Posts from Opiophile were gathered by identifying and downloading all posts with references to the products via customized software developed by Epidemico. Posts from Bluelight were gathered by creating and searching a copy of the forum’s underlying database in cooperation with the forum’s administrators. All posts containing references to the 3 antidepressants were subjected to customized natural language processing tools that identified formal and vernacular references to misuse-related Preferred Terms. The Preferred Terms were then associated with 1 of the 3 study drugs [38]. For this study we utilized the English Language Medical Dictionary for Regulatory Activities (MedDRA) version 18.0 terminology, including the broad scope Standardized MedDRA Query (SMQ) “Drug abuse, dependence, and withdrawal.” MedDRA is a clinically validated international medical terminology utilized by regulatory authorities throughout the drug lifecycle process. It is the international medical terminology developed under the auspices of the International Conference on Harmonisation of Technical Requirements for Registration of Pharmaceuticals for Human Use (ICH) [70]. In addition to this query, 3 Preferred Terms outside of this SMQ were added: Injection, Injection site reaction, and Legal problem (see Multimedia Appendix 1). All posts mentioning the target antidepressants were reviewed by a coding team composed of pharmacists, a physician, epidemiologists, Web-based harm-reduction forum administrators, and providers of health and social services to people who use drugs nonmedically (see Multimedia Appendix 2). Each post was evaluated by a single coder, and challenging posts were further reviewed by 1 or more additional members of the coding team. Coders extracted available information about authors’ expressed behaviors, intentions, experiences, and sociodemographic profile for posts that referenced misuse or nonmedical use of antidepressants (see Multimedia Appendix 3). The population was thus self-selecting and voluntary and may include users from any country or background as long as they posted in the English language and agreed to the site’s policies. In addition, the acquired data were stripped of personally identifiable identification (PII) and provided in a deidentified format.

### Definitions

Complete alignment is not apparent among regulators, the pharmaceutical industry, and harm reductions practitioners regarding how to define the misuse or nonmedical use of prescription products [71]. The World Health Organization states that the nonmedical use of a drug is considered “misuse,” whereas the FDA defines nonmedical use of a drug as “abuse” [72-74]. Abuse is a term that is widely used but varies in meaning. The term “abuse” sometimes conveys a negative connotation or denotes disapproval [75]. In the United States, the term generally refers to problems of psychoactive substance use for both prescription and nonprescription compounds (see Table 1).

In this study the SMQ Drug abuse, dependence, and withdrawal was utilized. Due to inherent regulatory commitments, the authors of this publication also opted to utilize the FDA definitions of abuse and misuse [73,74]. However, when referring to activities that fall under the FDA definition of abuse, we use the term “nonmedical use.” When the term “abuse” is used, it is not the intention of the authors to disapprove of or pass judgment on any person involved in substance use or the online communities where this use is discussed. In addition, the information and discussion presented here should not be viewed as suggesting or approving of the misuse or nonmedical use of these antidepressants.

**Table 1:** Definitions of abuse and misuse.

Agency	Definition of “abuse”	Definition of “misuse”
World Health Organization [72]	Persistent or sporadic excessive drug use inconsistent with or unrelated to acceptable medical practice.	Use of a substance for a purpose not consistent with legal or medical guidelines, as in the nonmedical use of prescription medications.
Food and Drug Administration [73,74]	The nonmedical use of a drug, repeatedly or even sporadically, for the positive psychoactive effects it produces.	The use of a drug outside label directions or in a way other than prescribed or directed by a health care practitioner. This definition includes patients using a drug for a condition different from that for which the drug is prescribed, patients taking more drug than prescribed or at different dosing intervals, and individuals using a drug not prescribed for them, although for therapeutic purposes.

### Manual Coding

Manual coding is the process of manually reviewing posts to extract medical insights, similar to chart abstraction in traditional studies. The coding team completed standardized training prior to evaluating posts for this project and met regularly to discuss challenging posts and determine standards. All decisions and guidance were tracked and documented in a coding manual, with updates added as new situations occurred and team decisions were made.

The FDA definitions of misuse and nonmedical use above were utilized to guide the coders. If a posting author described utilizing one of the drugs specifically for the potential psychoactive effects, the post was coded as “nonmedical use.” Alternatively, if a posting author described taking the drug for medical purposes, but outside of how it was prescribed or labeled, the post was coded as “misuse.”

The coding of posts was conducted utilizing a custom-built Web application called “In-sight Explorer,” which helped ensure that each reviewer was presented with a randomized set of forum posts to evaluate [76]. Additionally, the source of the posts was blinded to the coders. If a coder had any concern about how to answer any question relating to an individual post, the software helped to facilitate collaboration among the reviewers by enabling them to send a request for secondary review to any of the other coders or the whole group.

The software takes advantage of contextually highlighting the post content through the use of RxNorm- and MedDRA-controlled vocabularies to help coders quickly identify those portions of the text that may be relevant to the review process. RxNorm is a catalog of the standard names given to clinical drugs and drug delivery devices in the United States to enable efficient and accurate communication between electronic systems, independent of software and hardware capacity [77]. A screenshot of the coding tool is shown in Multimedia Appendix 4. All of the metadata collected about each post by manual coding is then recorded into a central database, which serves as the basis for results presented in this paper.

### Interrater Reliability (IRR)

Metrics of interrater agreement were calculated to assess the coding team’s agreement on tagging of posts. A random sample of 10 posts was gathered from the dataset and evaluated by all members of the coding team using the same questions and response options available in the manual coding interface. Agreement between coder-applied tags was then evaluated by calculating Fleiss kappa metrics of interrater agreement [78]. The use of Fleiss kappa was justified by the number of raters being assessed (11) and the nominal-scale format of ratings that were applied. The analyses included responses to the first two questions in the coding protocol, which asked coders to identify whether the post included reference to misuse or nonmedical use of in-scope products and what type of reference was made where applicable. Additional questions were omitted from analysis to reflect the coding protocol instruction to leave default answers unchanged if relevant information was not present in each post, thereby preventing artificial inflation of interrater agreement.

### Ethics

In this study, we analyzed the archives of two Web forums. Two main areas of ethical focus were considered for this work: informed consent from individuals and communities and the protection of PII.

We drew from the heuristic approach provided by McKee and Porter [79] that charts 2 dimensions against each other: private to public communication and sensitive to nonsensitive information. Content that is deemed sensitive and is in the public domain sits in a gray zone from an ethical perspective, and the extent of protection for the individuals who write the content and the communities that host the content should be assessed on a case-by-case basis. The community discussions demonstrate that contributors are aware of the public nature of the content that they post, and almost all contributors utilize pseudonyms to mask their identities. Although the subject matter may be seen as sensitive, these elements led the research authors to determine that consent from individual contributors was not necessary to conduct the research. It was also important to maintain any particular contributor’s anonymity, as the extent to which their pseudonym may reveal identifying information about them is unknown to the researchers. Therefore, to protect the identity of all post authors, PII was removed from all posts by a third-party vendor before receipt of the posts for coding. The types of PII removed included screen names, user names, first and last names, and addresses. In addition, where posts were included as examples in this paper, the post text has been paraphrased and altered in nonmeaningful ways to protect people’s identity and to prevent unmasking using Internet search engines. Because our research did not involve intervention or interaction with the individuals, nor is the information individually identifiable, our study did not meet the criteria of the Office for Human Research Protections (OHRP) framework that guides institutional review board (IRB) status. As such, IRB approval was not pursued.

Some researchers anonymize the names of the Web forums that they utilize as data in order to further assure confidentiality of the individual contributors or because the group had neither been actively involved in the research nor given consent to be involved [80,81]. Here, we took a participatory or partnership approach [82]. Bluelight has a research portal accessible from the front page of the website, which asserts Bluelight’s ownership of the forum content and instructs researchers to contact Bluelight administrators to discuss proposals for research, including archival analyses. The researchers contacted Bluelight to initiate discussions regarding this project, resulting in a partnership approach involving regular contact and contribution of Bluelight representatives to this study.

We contacted Opiophile via email to request consent and terms of access for gathering data from that forum. As no response was received from Opiophile, we reviewed the site’s privacy notice and user agreement and determined that gathering data for research purposes was within the scope of permitted uses. Opiophile forum posts were gathered using customized Web-crawling software that stored the primary body of text included in each post. Usernames, post titles, thread titles, or other information allowing retrospective identification of the authors’ Web-based identities were not included in the dataset used for coding or analysis.

We contacted a third potential data source, Erowid, to request consent and terms of access for gathering samples from their database of user-reported experiences with drugs. No response was received from Erowid, and their usage agreement explicitly prohibited data gathering or publishing of analyses without prior permission. In light of those policies and in the absence of response from site administrators, Erowid was excluded as a data source for this study.

## Results

### General Results

A total of 7270 posts were reviewed, containing 7756 references to at least one of the chosen products (ie, about 500 posts referenced more than 1 antidepressant). For purposes of simplicity, we refer to the 7756 as the denominator for proportion calculations. Of the total 7756 posts, 668 contain reference to misuse or nonmedical use of the product as defined above. This was 8.61% (668/7756) of the total reviewed, and within those 668 posts, 425 (63.6%, 425/668) were about nonmedical use and 243 (36.4%, 243/668) were about misuse (see Multimedia Appendix 5). The remainder of the posts made reference to in-scope products and drug use, but did not describe specific acts, intentions, or effects of nonmedical consumption. This nonmedical use and misuse subset of the data (n=668) was further analyzed as noted below. A breakdown across the 3 in-scope products is shown in Table 2. For demographic information including age, gender, country, ethnicity, and socioeconomic status, none of these was available for more than 4% of the posts.

In total, 656 (98.2%, 656/668) posts came from Bluelight and 12 (1.8%, 12/668) from Opiophile. The difference between Bluelight and Opiophile numbers may be due to Opiophile’s primary focus on opioids, periods when Opiophile was closed or down, length of time the 2 sites have been active (Bluelight since 1997 and Opiophile since 2003), and the difference in size between the 2 websites.

To better contextualize the overall numbers of these posts, the numbers of posts for controlled substances with nonmedical use potential are available for comparison in Table 3. Discussions for the noncontrolled substances (first 3 in Table 3) were considerably fewer than for controlled substances.

**Table 2:** Breakdown of posts among 3 antidepressants.

Posts		Bupropion	Amitriptyline	Venlafaxine	Total posts
Individual drug posts reviewed, n (%) of total posts reviewed		3472 (44.77)	1105 (14.25)	3179 (41)	7756
**Misuse or nonmedical use-related posts, n (%) of total drug specific posts**		438 (12.6)	100 (9.1)	130 (4.1)	668 (8.61)
	Nonmedical use posts^a^	305 (69.6)	60 (60)	60 (46.2)	425 (63.6)
	Misuse posts^a^	133 (30.4)	40 (40)	70 (53.9)	243 (36.4)

^a^If a post contained both a nonmedical use and misuse mention, it was captured as nonmedical use.

**Table 3:** Total number of posts for 7 different drugs.

Product	Bluelight	Opiophile	Total^a^
Bupropion	4058	39	4097
Amitriptyline	1183	6	1189
Venlafaxine	3508	19	3527
Methylphenidate	12,274	95	12,369
Alprazolam	41,334	835	42,169
Buprenorphine	44,639	1538	46,177
Oxycodone	104,270	2269	106,539

^a^Total numbers before any removal of duplicates or manual review of posts; thus different from the final product numbers for in-scope products presented above for the most appropriate comparisons to be made.

### Misuse and Nonmedical Use Data Subset Results

Additional characteristics of each post were examined by the coding team. The results from each characteristic or data point extracted are shown in Multimedia Appendix 6, with paraphrased example posts for illustration.

**Figure 1:**
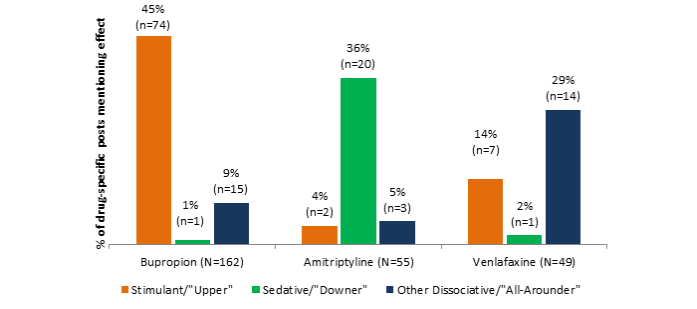
Desired effect posts by drug.

Information about the desired effect of a drug was deduced from 266 total posts (39.8%, 266/ 668 post dataset). Figure 1 shows that although all 3 pharmaceuticals have the same antidepressant indications, their desired effects in nonmedical use are quite different. Bupropion seems to be most desired as a stimulant, whereas amitriptyline most desired as a sedative, and venlafaxine as a dissociative.

Example “desired effect” posts:

I dissolved a 150mg bupropian in warm water. Then I put it in the freezer and took a nap. The solution had frozen, but it thawed quickly upon shaking. I injected the solution into my arm after filtering twice through filters I got from needle exchange. Is it possible the bupropion was altered by either the freezing, or the boiling? I am definitely feeling stimulated, else I wouldn't be bothering to post and I'd continue lurking.

I want to get more sedation without upping my benzos or opiates. Can I add Elavil to the mix, or maybe take something out of the mix and add amitriptyline since I know it is sedating.

For me, going into rehab didn't stop me from finding drugs. I pretended to do well in rehab to get out. I was buddies with the nurse in rehab and he got me meth. I took my mom’s prescription pad and wrote venlafaxine to induce mania, got her to prescribe me Ritalin, etc. Rehabilitation never ends.

**Figure 2:**
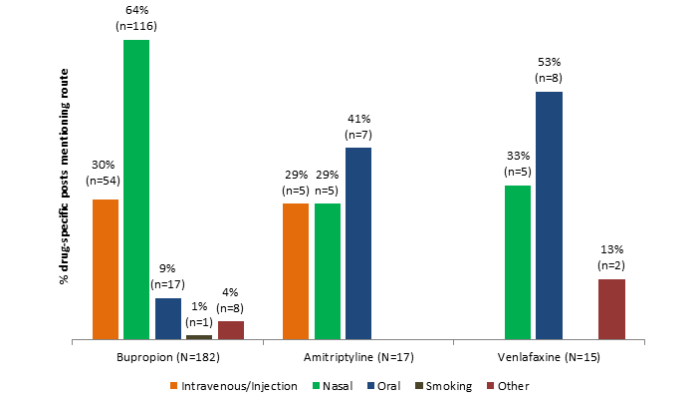
Route of administration details by drug.

Information about route of administration for nonmedical use was deduced from 214 total posts (32%, 214/ 668 post dataset), with bupropion accounting for 182 posts (41.6 %, 182/438 of bupropion posts), amitriptyline for 17 posts (17%, 17/100 of amitriptyline posts), and venlafaxine for 15 posts (11.5%, 15/130 of venlafaxine posts). Of note, 21 bupropion posts mentioned more than 1 route to equal 196 route mentions within the 182 bupropion posts. Figure 2 shows that the preferred nonmedical route of administration for bupropion is intranasal followed by intravenous or injection. The “other” category includes plugging, rectal, parachuting, foiling, and “abusing any other way.”

Example “route of administration” posts:

I scared myself to death once. Didn't have anything and I had heard that bupropians could be snorted for a high and I did, man that stuff hits you faster than cocaine...even numbs you the same. But it feels like your snorting knives...never again!

I was dumb enough once to snort amitriptyline which is a great benzo and opiate activator. I guess I wanted a quick onset, instead I got 30 minutes of awful burning pain! I've only had light blue and yellow pills without any markings, I don't know if the brand name burns as much but I've learned my lesson now.

Maybe I scored with my Effexor prescription if I took more. I took several 75mg tablets at one time and did feel more alert, happy. I can take them as prescribed and benefit from the effect it has on making methadone more effective or I can use the month's supply in a few days and get a great high.

**Figure 3:**
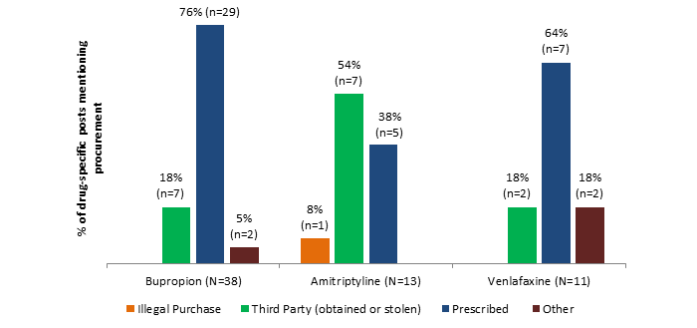
Method of procurement details by drug.

The means of procurement of the drug are shown in Figure 3. Overall, procurement method was mentioned in 62 (9.3%, 62/668) of the posts, with bupropion accounting for 38 (8.7%, 38/438 drug specific posts), amitriptyline for 13 (13%, 13/100 drug specific posts), and venlafaxine for 11 (8.5%, 11/130 drug specific posts). Although they have similar licensed indications, the 3 drugs show some differences in most common route of procurement, with amitriptyline showing a higher propensity for procurement via stealing or illegal purchase than bupropion or venlafaxine, which were most commonly acquired via prescription from a health care provider. The “other” category comprised implied pharmacy dispensing error, “found on ground,” “by accident,” and “came across.”

Example of “method of procurement” posts:

I have had some bupropion around from an old prescription. I had read about people snorting it in various forums. The reviews were more negative than positive and the positive seemed really weak to say the least. But I was bored one day after drinking a few beers and smoking weed and I thought that it won't hurt 1 time. I was wrong, the experience was turned bad after a while. I snorted 500 mg over the course of about 5 hours. The only positive sensation was somewhat more alert at first and having a buzz.

My friend found a lot of amitriptyline so I’m wondering...does it have potential for recreational purposes if used with weed? What about starting dose, good recreational dose, and dangerous dose? Would codeine or valium be a good addition?

Last week, I found some venlafaxine pills on the street...Anyone else taken these while drinking and have an intense experience?

### Interrater Reliability Results (IRR)

The Fleiss kappa for interrater agreement among 20 items with 7 categorical response options evaluated by all 11 raters was 0.448 (95% CI 0.421-0.457).

## Discussion

### Principal Findings

This paper makes 2 contributions. First, we were able to design a methodology that detects misuse and nonmedical use of noncontrolled substances in harm-reduction Web forums, a novel pharmacovigilance process. Second, we were able to validate this methodology by confirming the formerly sparse literature and spontaneous adverse reports regarding the nonmedical use of bupropion, previously unconfirmed by the DAWN database study. The evidence from these forums suggests that despite being noncontrolled substances, these 3 antidepressants have properties sought out by those seeking positive psychoactive effects. Collectively, the data reveal that nonmedical use of the 3 antidepressants differs markedly. The most commonly discussed types of effects were stimulant for bupropion, sedative for amitriptyline, and dissociative for venlafaxine. This is consistent with what is seen in literature reports for each drug, respectively. The data also indicate that amitriptyline was sought most frequently in combination with other (usually controlled) substances for euphoric effect; such a combination was reported much less frequently for bupropion and venlafaxine. Nasal insufflation was the most popular route for abuse of bupropion, seen nearly twice as often as with either comparator drug. One of the most interesting findings overall was the high percentage of posts in these forums which actually discouraged the nonmedical use of bupropion, whereas posts encouraging nonmedical use were relatively constant across the 3 drugs. Nonmedical use was discouraged either owing to side effect profile (nasal burning), danger (risk of seizure), or failure to achieve the desired effect by various post authors. These data also provide a rare glimpse into the combinations of antidepressants and other substances that are used outside of a medical context, providing a basis for harm-reduction messaging.

### Methodology Strengths

This novel methodology allows for the differentiation between misuse and nonmedical use patterns among 3 commonly prescribed antidepressants, all of which are noncontrolled substances according to international treaties. This study reveals that the posted experiences of antidepressants when misused or used nonmedically are heterogeneous. Whereas animal studies have suggested some of these differences, rodent models are limited in their ability to discern certain mammalian effects, such as dissociative effects, sought out by nonmedical users. In addition, epidemiologic surveillance systems have not had the product-level resolution to discern the subjective differences, often combining all medications in this therapeutic area into 1 category. The methodology provided herein suggests that Web forums may be able to fill this key information gap.

The medications selected for this evaluation are antidepressants, noncontrolled substances that have limited epidemiologic surveillance for misuse and nonmedical use. Lingering questions about antidepressant misuse and nonmedical use typically are not captured and measured by most large-scale epidemiologic surveys, making it challenging to characterize from both a drug misuse and a toxicological perspective [3]. The social listening methodology outlined in this paper provides a framework for further exploration of the misuse and nonmedical use of other noncontrolled substances.

The “controlled substance” classification is used in accordance with international treaties to designate drugs (both medicinal and illicitly manufactured) that have shown potential for abuse [83]. The World Health Organization’s Expert Committee on Drug Dependence (WHO-ECDD) makes scientific recommendations that are codified into international treaty obligations by the International Narcotics Control Board (INCB) in Vienna, Austria. In order to conduct this work, these multilateral bodies make extensive use of surveillance systems across member states [84]. Although many privately and publicly sponsored surveillance systems exist for pharmaceutical and illicitly manufactured controlled substances [85-90], there is a paucity of information about the nonmedical use of noncontrolled substances. Social listening may potentially drive validation or rejection of already existing hypothesis generating data sources utilized in traditional safety surveillance that on their own may contain small numbers or missing information (spontaneous reports, literature, surveys). This may be particularly fruitful with challenging areas of surveillance, such as the nonmedical use of noncontrolled drugs, as presented here.

This is an entirely new data source and method of data collection for pharmacovigilance activities. This dataset provided much more detail than traditional forms of pharmacovigilance data sources and holds great potential, especially in areas such as the nonmedical use of noncontrolled substances, where data have been difficult to obtain through standard pharmacovigilance practices. The unscripted and unsolicited format of the data provides an understanding of the thoughts of people who use these drugs, in their own words. The longevity of these forums to date may provide an ongoing means of monitoring the extent of abuse of various drugs.

### Methodology Limitations

It is unclear how representative the experiences of those who post anonymously on the Web are of those of the general nonmedical drug-using population and nonmedical use of bupropion in particular, and prevalence of drug use in the greater population cannot be extrapolated from these data. Access to Internet connections, literacy, and social norms for public discussion on drug nonmedical use vary considerably around the world. In addition, there may be a bias toward younger age groups, who are “digital natives” and have more plasticity with their Web-based identities. For example, in a survey of 897 Bluelight members in 2012, the mean age was 25 years and 76% were male [34]. The representativeness of posts is also compromised by the “1% rule,” which states that 1% of a Web community posts the vast majority of content (“superusers”), whereas another 9% is posted by “contributors,” and 90% do not post content at all (“lurkers”). This concept has been recently confirmed [91]. Although there are clear differences in the experiences, uses, and perceptions of the antidepressants studied here, limitations in representativeness could compromise attempts to extrapolate prevalence from the quantification of post content.

There are other limitations with this methodology. In order to protect privacy, the identity of authors was masked to the researchers. As a result, it is not known how many post authors are represented in the 7270 posts. However, maintaining multiple accounts over time is a practice endorsed by long-term users. Even with transparency into author pseudonyms, we still would not gain insights into a direct 1:1 ratio of authors and pseudonyms. Also limiting is the inherent scale limitations associated with manual coding, which can be labor and time intensive. For larger or ongoing projects, computational techniques should be considered. Finally, this is a novel methodology and the weight of evidence of a social media study and where it sits in the hierarchy of evidence still needs to be formally assessed and determined.

Bluelight is considered to be the largest repository for discussions about nonmedical use and misuse of substances. At the time of writing (September 13, 2016), Bluelight had a more popular global ranking than other drug discussion websites, such as Drugs-forum, Opiophile, and Erowid, based on the traffic ranking at Alexa, which was “calculated using a combination of average daily visitors to the site and pageviews on the site over the past 3 months. The site with the highest combination of visitors and pageviews was ranked “#1” [92]. Therefore, we believe this lends credibility of confidence to our work.

A common approach among researchers who analyze the content of publicly available Web forum communities is to copy the available data and produce their research independently [80,81,93-95]. Some researchers may believe that the community itself would not welcome research collaborations. They may also be unaware of intellectual property implications, applicable copyright laws, or terms of service specific to these communities which prohibit noncollaborative practices, such as unauthorized mining or harvesting of data, often referred to as “scraping.” Bluelight’s experience has been that many researchers simply do not consider the Bluelight community leaders as key stakeholders in their research: researchers may describe concerns about the ethics of engaging with individual contributors, but often appear unaware about the forum community’s broader interest in how the community is contributing to scientific knowledge and is represented by researchers.

The collaborative or participatory approach described here and previously published [82] is an alternative ethical framework that has a number of advantages. First, the researchers can identify the Web community in publications confidently, providing greater transparency and context for their findings. Second, Web community representatives can be engaged in the research process, working alongside researchers to help interpret and contextualize emergent understandings arising from the research. For example, in this study, it was helpful when community representatives resolved ambiguities arising from commonly used expressions and identified PII, such as pseudonyms embedded in text, that were not completely obvious to community outsiders. Third, researchers taking the collaborative approach can be confident that they are not breaching the terms of service or intellectual property rights of Web communities because the dataset was obtained directly and with authorization from community leaders. While not all Web communities welcome this kind of collaboration, the authors of this paper believe it is important for researchers who wish to utilize community data to attempt to engage communities in the first instance, rather than assume a lack of interest or capacity from the outset.

### Conclusions

This study demonstrates the potential impact of anonymous conversations in Web-based harm-reduction forums, where safe communities are created for the exchange of ideas and experiences regarding drug use. Our experience suggests benefit from collaboration directly with Web forum communities. The evaluation of 2 harm-reduction forums across 3 antidepressants revealed a level of misuse and nonmedical use detail not seen in traditional surveillance data sources and confirmed previous observations for bupropion. Particular insights were seen in identifying and characterizing desired effects of misuse and nonmedical use, routes of administration for nonmedical use, and methods of drug procurement. In addition, the majority of forum posters discouraged the misuse and nonmedical use of all 3 antidepressants. Although social media listening is a promising data source for pharmacovigilance, concerns remain around the generalizability of these results and the value for clinicians and regulatory agencies. Despite these limitations, it warrants noting that this study captured detailed data around the historically difficult-to-monitor area of misuse and nonmedical use of noncontrolled substances. Further study is needed to establish the benefits and limitations of social media listening in this area of safety surveillance.

## Appendix

Multimedia Appendix 1 Study Preferred Terms.

Multimedia Appendix 2 Number of posts per individual coder.

Multimedia Appendix 3 Criteria evaluated during coding.

Multimedia Appendix 4 Insight Explorer for manual coding.

Multimedia Appendix 5 Example nonmedical use and misuse posts.

Multimedia Appendix 6 Further details of posts extracted by manual coders with example posts.

## Abbreviations

DAWN: Drug Abuse Warning Network
INCB: International Narcotics Control Board
IRB: institutional review board
IRR: interrater reliability
MedDRA: Medical Dictionary for Regulatory Activities
NSDUH: National Survey on Drug Use and Health
OHRP: Office for Human Research Protections
PII: personally identifiable identification
PTs: preferred terms
RADARS: Researched Abuse, Diversion and Addiction-Related Surveillance
SMQ: Standardised MedDRA Query
SNRI: serotonin and norepinephrine reuptake inhibitor
TCA: tricyclic antidepressant
FDA: Food and Drug Association
WHO: World Health Organization
WHO-ECDD: World Health Organization’s Expert Committee on Drug Dependence
