# Supplementary material for: Using Social Listening Data to Monitor Misuse and Nonmedical Use of Bupropion: A Content Analysis
Source: JMIR Public Health Surveill. 2017 Feb 1;3(1):e6. doi: 10.2196/publichealth.6174 (PMC5311422; doi:10.2196/publichealth.6174)
Supplement: Multimedia Appendix 1 [file publichealth_v3i1e6_app1.pdf]

Accidental overdose	Intentional overdose
Dependence	Intentional product misuse
Disturbance in social behaviour	Intentional product use issue
Dopamine dysregulation syndrome	Maternal use of illicit drugs
Drug abuse	Medication overuse headache
Drug abuser	Narcotic bowel syndrome
Drug administered at inappropriate site	Needle track marks
Drug dependence	Neonatal complications of substance abuse
Drug dependence, antepartum	Overdose
Drug dependence, postpartum	Polysubstance dependence
Drug detoxification	Prescribed overdose
Drug diversion	Prescription form tampering
Drug level above therapeutic	Rebound effect
Drug level increased	Steroid withdrawal syndrome
Drug rehabilitation	Substance abuse
Drug screen	Substance abuser
Drug screen positive	Substance use
Drug tolerance	Substance-induced mood disorder
Drug tolerance decreased	Substance-induced psychotic disorder
Drug tolerance increased	Toxicity to various agents
Drug withdrawal convulsions	Withdrawal arrhythmia
Drug withdrawal headache	Withdrawal syndrome
Drug withdrawal maintenance therapy	Legal problem
Drug withdrawal syndrome	Injection site reaction
Drug withdrawal syndrome neonatal	Injection
