# Supplementary material for: Using Social Listening Data to Monitor Misuse and Nonmedical Use of Bupropion: A Content Analysis
Source: JMIR Public Health Surveill. 2017 Feb 1;3(1):e6. doi: 10.2196/publichealth.6174 (PMC5311422; doi:10.2196/publichealth.6174)
Supplement: Multimedia Appendix 2 [file publichealth_v3i1e6_app2.pdf]

Coder	Number of posts (N=7270)
Coder 1	254
Coder 2	198
Coder 3	2225
Coder 4	194
Coder 5	2102
Coder 6	281
Coder 7	174
Coder 8	590
Coder 9	200
Coder 10	204
Coder 11	848
