# Supplementary material for: Using Social Listening Data to Monitor Misuse and Nonmedical Use of Bupropion: A Content Analysis
Source: JMIR Public Health Surveill. 2017 Feb 1;3(1):e6. doi: 10.2196/publichealth.6174 (PMC5311422; doi:10.2196/publichealth.6174)
Supplement: Multimedia Appendix 3 [file publichealth_v3i1e6_app3.pdf]

Is the post describing misuse or nonmedical use of the in-scope product(s)?
What is the category of this post? (misuse, nonmedical use, other)
Poster age range, gender, location, ethnicity
Route of administration
Length of use
Dosage
Is the poster encouraging or discouraging of the misuse or nonmedical use?
Procurement method
Desired effect from misuse or nonmedical use
Misuse or nonmedical use outcome or effect
Socioeconomic status
Is the post author seeking information?
Drugs combined for misuse or nonmedical use
Magnitude of misuse or nonmedical use within the community
Misuse or nonmedical use happening within the criminal justice system
