# Supplementary material for: Using Social Listening Data to Monitor Misuse and Nonmedical Use of Bupropion: A Content Analysis
Source: JMIR Public Health Surveill. 2017 Feb 1;3(1):e6. doi: 10.2196/publichealth.6174 (PMC5311422; doi:10.2196/publichealth.6174)
Supplement: Multimedia Appendix 4 [file publichealth_v3i1e6_app4.pdf]

Insight Explorer

Insight User

Home | Drug Abuse | Locked Posts | Admin Tools | Logout

Review New Post

Please note that while this post is open, no one else may edit it. Please complete the review before closing the window.

SOURCE	POST ID	POST DATE	COUNTRY	IND SCORE	PROTO-AE/MENTION	IME LISTED	REVIEWED
Web Forum	1234	Dec 01, 2015	Unknown	0.5523	Mention	No	Yes

TAGGED PRODUCTS		TAGGED AES
wellbutrin	Maps to Generic:	nonspecific reaction
amphetamines	Drug Not Mapped	**prescription form tampering**
adderall	Maps to Generic:	
amphetamine	Maps to Brand:	

POST CATEGORIZATION

Is this an abuse/misuse-related post or other post of interest for an in-scope product?	Yes
What is the category of this post?	Abuse
Exclude post due to spam or advertisement?	No

Post Content

Were you taking **adderall**? Using more than 100mg over this short amount of time is a LOT. **wellbutrin** can be a substitute for **amphetamine** and has some stimulant effect. Use with care.

Poster Information

Patient Age Range:	Unknown
Patient Gender:	Unknown
Poster Location:	
Set Country:	Unknown

Product: wellbutrin [In Scope]

Route:	Nasal insufflations (e.g., snorting)
Length of Use:	
Dosage:	
Abuse endorsing or discouraging?	Abuse - Discouraging
Procurement Method:	Unknown
Desired Effect:	Unspecified high
Desired effect similar to:	
Misuse/abuse outcome or effect:	increase side effects as we

Post Mentions

Contains PII:	No	Drugs combined for abuse:	Yes
Socioeconomic Status:	No	Community Mention:	No
Ethnicity:	No	Happened within criminal justice system:	No
Seeking Info:	No	Include verbatim text:	Yes

Any additional comments:

Would you like to have someone else review this post?

Secondary Reviewer: None

Save Review | Cancel Review
