# Supplementary material for: Using Social Listening Data to Monitor Misuse and Nonmedical Use of Bupropion: A Content Analysis
Source: JMIR Public Health Surveill. 2017 Feb 1;3(1):e6. doi: 10.2196/publichealth.6174 (PMC5311422; doi:10.2196/publichealth.6174)
Supplement: Multimedia Appendix 5 [file publichealth_v3i1e6_app5.pdf]

Example ‘nonmedical use’[a,b]	Example ‘misuse’[a,b]
I read an article online about a new kick in Chicago and other cities about the injection of Wellbutrin—it is referred to as the poor man’s cocaine. Tried it, and had a nice euphoria with increased energy.	*I am prescribed an antipsychotic, something for anxiety, and bupropion. I can have fun of it i just have to skip the bups and if i'm on them the anti-psychotics.*
We have been insufflating (snorting) bups. But this has been extremely painful and Im worried its damaging my sinus/nasal cavity. I have one friend who injects them but that is not an option for the rest of us.Is there a better way to take these and still feel an effect?	*I have also been scripted Effexor—don’t know why. I am a daily marijuana smoker and enjoy using other drugs on a recreationally. Everything from cocaine to xanax. I may want to take a scrip holiday—any thoughts on withdrawal?*
Maybe effex? it has some opiod actions and it should be easy to get.	*I've got some amitrip tablets, using them to try and help me sleep, but i cant stay asleep for longer than a few hours after taking one, and have light sleep after that, waking up all the time. I was wondering if it would be safe to take 2 or 3 of them at once to try and stay asleep for longer?*

[a]Posts have been modified in non-meaningful ways to protect the identity of the author.
[b]Posts were coded as nonmedical use or misuse regardless of if the action was only discussed or actually occurred.
