# Supplementary material for: Using Social Listening Data to Monitor Misuse and Nonmedical Use of Bupropion: A Content Analysis
Source: JMIR Public Health Surveill. 2017 Feb 1;3(1):e6. doi: 10.2196/publichealth.6174 (PMC5311422; doi:10.2196/publichealth.6174)
Supplement: Multimedia Appendix 6 [file publichealth_v3i1e6_app6.pdf]

	Bupropion (% of total Bupropion posts, N=438)	Amitriptyline (% of total Amitriptyline posts, N=100)	Venlafaxine (% of total Venlafaxine posts, N=130)	Total (% of total misuse/nonmedical use posts, N=668)
**Dosage information known**	123 (28%)	35 (35%)	31 (24%)	189 (28%)
I've been using 75mg of venlafaxine, 25mg 5-htp, and 150mg bupropion twice daily the last few days. I took those things maybe 5 hours ago. An hour later I used 175mg …				
**Nonmedical use-encouraging**	54 (12%)	10 (10%)	14 (11%)	78 (12%)
… maybe like a combo of cocaine and adderall without the paranoia or hot flashes..... Did find myself wanting to do it again and try increased dose….. Try it once yourself and see….				
**Nonmedical use-discouraging**	178 (41%)	22 (22%)	24 (18%)	224 (34%)
"I am sharing what happened so other won't do this. I took 800 mg and ….I went into convulsions! I could have died. Please PLEASE don't ever abuse Antidepressants"				
**Neither nonmedical use-discouraging or encouraging-- a classification of "neither" was assigned if a post was both encouraging and discouraging**	68 (16%)	19 (19%)	28 (22%)	115 (17%)
*bupropion is sometimes referred to as crack so I would strongly suggest not mixing the two. This could cause seizure. However, you can have effects from snorting—it burns like h***. People have told me its better than adderal and they have abused it for a long time.*				
**Procurement method mentioned (obtained or stolen from third party, illegal purchase, prescribed by health care provider, other)**	38 (9%)	13 (13%)	11 (8%)	62 (9%)
I got some venlafaxine from one friend who used it for fun a lot…				
**Drugs combined for misuse/nonmedical use**	72 (16%)	40 (40%)	27 (21%)	139 (21%)
…haven't tried amitriptyline before but I got access to 75mg pills, that I would probably cut in half or something since i've never done before. I know they're for sleep, but what if I mixed with a cocktail of kpin and cipralex/lexapro......				
**Mention of magnitude of misuse/nonmedical**	112 (26%)	17 (17%)	11 (8%)	140 (21%)

**use within community**				
Hopeful that someone experienced with this might reply. It's becoming quite popular in my area. My drug of choice is crack, but these pills [bup] are cheap & easy to come by…				
**Mention of use within the criminal justice system**	19 (4%)	4 (4%)	0 (0%)	23 (3%)
another drug abused in the big house is Wellbutrin (bupropion), snorted for its stimulant effects…				
